# Genome sequences of four virulent *Klebsiella pneumoniae* phages targeting multidrug-resistant clinical isolates from Ethiopia

**DOI:** 10.1128/mra.01213-25

**Published:** 2026-06-11

**Authors:** Assefa Asnakew Abebe, Molalegne Bitew, Alemayehu Godana Birhanu, Dawit Hailu Alemayehu, Abaysew Ayele, Keyru Tuki, Yimer Demssie, Lanchisl Tsegaye Mulualem, Tesfaye Sisay Tessema

**Affiliations:** 1 Biotechnology Research Center, Addis Ababa University 37602, Addis Ababa, Ethiopia; 2 Unit of Molecular Biology, Department of Medical Laboratory Sciences, Institute of Health, Bule Hora University 435723, Bule Hora, Ethiopia; 3 Bio and Emerging Technology Institute (BETin) 435723, Addis Ababa, Ethiopia; 4 Amauer Hansen Research Institute, Jumma Road ALERT Compound, Addis Ababa, Ethiopia; 5 Department of Midwifery, Institute of Health, Bule Hora University, Bule Hora, Ethiopia; University of Pittsburgh School of Medicine 12317 , Pittsburgh, Pennsylvania, USA

**Keywords:** phage therapy, *Klebsiella pneumoniae*, multidrug-resistant, genome analysis

## Abstract

We present genome sequences of four lytic bacteriophages targeting multidrug-resistant (MDR) *Klebsiella pneumoniae* isolates. The bacteriophages belong to the genera *Taipeivirus*, *Drulisvirus*, *Przondovirus*, and *Webervirus*. These phages exhibit promising lytic activity against MDR *K. pneumoniae* clinical isolates. The genomes of the bacteriophages ranged in size from 15,773 to 166,437 bp.

## ANNOUNCEMENT

In light of the escalating issue of antimicrobial resistance, multidrug-resistant (MDR) *Klebsiella pneumoniae* represents a substantial public health concern (1, 2). The emergence of MDR strains, particularly those producing extended-spectrum beta-lactamases and carbapenemases, has exacerbated the challenge of treating infections caused by this organism (3). An alternative therapy being explored is phage therapy (4), which utilizes bacteriophages (bacteria-infecting viruses) (5). Phage therapy emerges as a promising alternative to combat these pathogens, offering potential solutions for effective treatment in the face of rising antimicrobial resistance (AMR) challenges (6–8). This study reports the genome sequence of four virulent *Klebsiella pneumoniae* phages from Ethiopia.

Bacteriophages were isolated from environmental samples comprising two river water samples and two hospital sewage samples (Table 1). Samples were screened against multidrug-resistant *K. pneumoniae* clinical isolates (Table 1). The characterization and AMR profile of the bacteria isolated and used in this study are reported by Abebe et al. (9). Details of isolation and biological characterization of the phages are provided in our previous publication (10). Spot assay was used for phage isolation, and streak plating and agar overlay assay were used for phage purification as described by Abebe et al. (10, 11). The phage lysates were treated with DNase and RNase (1 h, 37°C) to degrade extracellular nucleic acids, followed by enzyme inactivation with 20 mM ethylenediaminetetraacetic acid (EDTA). Proteinase K digestion (3 mg/mL) was performed at 56°C for 90 min, followed by phage DNA purification using the EZ-10 Spin Column Bacterial Genomic DNA Miniprep Kit (Omega Bio-tek, USA). DNA libraries were constructed using the Illumina Nextera XT DNA Kit according to the manufacturer’s protocol and sequenced in paired-end mode (2 × 150 bp) on the Illumina NextSeq 550 platform. The quality of raw sequencing reads was assessed using FastQC (v0.12.0) (12), and low-quality bases and adapters were trimmed using fastp (v0.23.0) (13), with a minimum quality threshold of Q20 and reads shorter than 50 bp removed after trimming. The quality of raw sequencing reads was assessed using FastQC (v0.12.0), and low-quality bases and adapters were trimmed using fastp (v0.23.0), with a minimum quality threshold of Q20 and reads shorter than 50 bp removed after trimming with a minimum quality threshold of Q20 and reads shorter than 50 bp removed after trimming. Taxonomic classification was performed using Kraken2 (v2.1.2) (14) to categorize reads at the genus and species levels and to filter out potential contaminants. *De novo* assembly was performed with MEGAHIT (v1.2.9) (15), and contigs from each sample were scaffolded to improve assembly quality. QUAST (v5.0.2) (16) was used for assembly quality assessment, while CheckV (v1.5.0) (17) was used to evaluate genome completeness. Phage-specific downstream bioinformatics analysis, including completeness assessments, lifestyle predictions, structural gene annotations, and taxonomic classification, was performed using PhageScope (18).

We identified four promising lytic bacteriophages KFM-TASP06, TTM-TASP117, BYM-TASP37, and MHM-TASP32 that show strong potential for phage therapy against MDR *K. pneumoniae* infections. Their effectiveness is supported by their lytic activity and the absence of undesirable genes in their genomes.

**TABLE 1:** Sequence analysis ummary of sequenced bacteriophages

Phage name	*K. pneumoniae* host	GenBank accession number	SRA accession number	Source	#Reads	#Contigs	Genome size (bp)	Genes	Completeness	GC content (%)	Genus	Life style	Similar phages
KFM-TASP06	TASP06	PV588648	SRS26965073	River	36,679	335	166,437	225	Complete	44.25	Taipeivirus	Virulent	Klebsiella phage phi1_146041 (PP889490)
TTM-TASP117	TASP117	PV588649	SRS26965074	Sewage	30,407	268	167,792	227	Complete	44.87	Taipeivirus	Virulent	Klebsiella phage XDRpn-1 (PP792980)
BYM-TASP37	TASP37	PV588652	SRS26965077	River	218,282	487	17,666	26	Partial	44.50	Drulisvirus	Virulent	Klebsiella phage RCIP0031 (OR532825)
MHM-TASP32	TASP32	PV588654	SRS26965079	Sewage	130,065	52	38,888	51	Complete	52.78	Przondovirus	Virulent	Klebsiella phage 066046 (MW042806)

## Data Availability

The genome sequences of the phages are available in the National Center for Biotechnology Information (NCBI) GenBank with accession numbers PV588648, PV588649, PV588650, PV588651, PV588652, PV588653, and PV588654. The raw sequence data have been deposited in the NCBI Sequence Read Archive (SRA) under BioSample accession numbers SAMN53043033, SAMN53043034, SAMN53043035, SAMN53043036, SAMN53043037, and SAMN53043038. These BioSamples are associated with BioProject PRJNA1355480 in the NCBI database.

## References

[1] Effah CY, Sun T, Liu S, Wu Y. 2020. Klebsiella pneumoniae: an increasing threat to public health. Ann Clin Microbiol Antimicrob 19:1. doi:10.1186/s12941-019-0343-8 31918737 PMC7050612

[2] Russo TA, Marr CM. 2019. Hypervirulent Klebsiella pneumoniae. Clin Microbiol Rev 32:10. doi:10.1128/CMR.00001-19 PMC6589860 31092506

[3] Imtiaz W, Syed Z, Rafaque Z, Andrews SC, Dasti JI. 2021. Analysis of antibiotic resistance and virulence traits (genetic and phenotypic) in Klebsiella pneumoniae clinical isolates from Pakistan: identification of significant levels of carbapenem and colistin resistance. Infect Drug Resist 14:227–236. doi:10.2147/IDR.S293290 33531820 PMC7846821

[4] Brives C, Pourraz J. 2020. Phage therapy as a potential solution in the fight against AMR: obstacles and possible futures. Palgrave Commun 6:1–11. doi:10.1057/s41599-020-0478-4

[5] Abedon ST, García P, Mullany P, Aminov R. 2017. Phage therapy: past, present and future. Front Microbiol 8:981. doi:10.3389/fmicb.2017.00981 28663740 PMC5471325

[6] Gordillo Altamirano FL, Barr JJ. 2019. Phage therapy in the Postantibiotic era. Clin Microbiol Rev 32:10. doi:10.1128/CMR.00066-18 PMC6431132 30651225

[7] Anand T, Virmani N, Kumar S, Mohanty AK, Pavulraj S, Bera BC, Vaid RK, Ahlawat U, Tripathi BN. 2020. Phage therapy for treatment of virulent Klebsiella pneumoniae infection in a mouse model. J Glob Antimicrob Resist 21:34–41. doi:10.1016/j.jgar.2019.09.018 31604128

[8] Herridge WP, Shibu P, O’Shea J, Brook TC, Hoyles L. 2020. Bacteriophages of Klebsiella spp., their diversity and potential therapeutic uses. J Med Microbiol 69:176–194. doi:10.1099/jmm.0.001141 31976857 PMC7431098

[9] Abebe AA, Birhanu AG, Tessema TS. 2026. Molecular characterization of virulence and antibiotic resistance genes in Klebsiella pneumoniae isolated from sputum samples at a tertiary hospital in Ethiopia. Sci Rep 16:8541. doi:10.1038/s41598-026-39069-3 41680374 PMC12976296

[10] Abebe AA, Birhanu AG, Tessema TS. 2026. Isolation and characterization of lytic bacteriophages with therapeutic potential against multidrug resistant Klebsiella pneumoniae from Ethiopia. Scientific Reports. 10.1038/s41598-026-39153-8 PMC12957310 41663479

[11] Abebe AA, Birhanu AG, Tessema TS. 2025. Isolation, purification, and phenotypic characterization of virulent Klebsiella pneumoniae phages from environmental samples in Addis Ababa, Ethiopia: a synergistic approach combining spot assay and streak plating. PLoS One 20:e0331955. doi:10.1371/journal.pone.0331955 40991621 PMC12459788

[12] Han SJ, Kim SK, Hong SM. 2024. Next-generation sequencing of microRNA in acquired middle ear cholesteatoma. Laryngoscope 134:4374–4382. doi:10.1002/lary.31507 38775212

[13] Chen S, Zhou Y, Chen Y, Gu J. 2018. Fastp: an ultra-fast all-in-one fastq preprocessor. Bioinformatics 34:i884–i890. doi:10.1093/bioinformatics/bty560 30423086 PMC6129281

[14] Wood DE, Lu J, Langmead B. 2019. Improved metagenomic analysis with kraken 2. Genome Biol 20:257. doi:10.1186/s13059-019-1891-0 31779668 PMC6883579

[15] Li D, Liu C-M, Luo R, Sadakane K, Lam T-W. 2015. Megahit: an ultra-fast single-node solution for large and complex metagenomics assembly via succinct de bruijn graph. Bioinformatics 31:1674–1676. doi:10.1093/bioinformatics/btv033 25609793

[16] Gurevich A, Saveliev V, Vyahhi N, Tesler G. 2013. Quast: quality assessment tool for genome assemblies. Bioinformatics 29:1072–1075. doi:10.1093/bioinformatics/btt086 23422339 PMC3624806

[17] Nayfach S, Camargo AP, Schulz F, Eloe-Fadrosh E, Roux S, Kyrpides NC. 2021. Checkv assesses the quality and completeness of metagenome-assembled viral genomes. Nat Biotechnol 39:578–585. doi:10.1038/s41587-020-00774-7 33349699 PMC8116208

[18] Wang RH, Yang S, Liu Z, Zhang Y, Wang X, Xu Z, Wang J, Li SC. 2024. Phagescope: a well-annotated bacteriophage database with automatic analyses and visualizations. Nucleic Acids Res 52:D756–D761. doi:10.1093/nar/gkad979 37904614 PMC10767790

